# Enhanced Transport and Permeation of a Polymeric Nanocarrier across the Retina by Mixing with ATP upon Intravitreal Injection

**DOI:** 10.3390/pharmaceutics13040463

**Published:** 2021-03-29

**Authors:** Kiyoon Kwon, Youngmin Hwang, Junyoung Jung, Giyoong Tae

**Affiliations:** School of Materials Science and Engineering, Gwangju Institute of Science and Technology (GIST), Gwangju 61005, Korea; kkykr333@gist.ac.kr (K.K.); ymhwang@gm.gist.ac.kr (Y.H.); mickey8444@gm.gist.ac.kr (J.J.)

**Keywords:** retinal pigment epithelium, pluronic, penetration, P2Y receptor

## Abstract

The outer part of the retina pigment epithelium (RPE) in the retina is the main site of neovascularization associated with retinal diseases. However, various obstacles interrupt the delivery of medicines across the RPE, mainly due to the well-developed tight junctions in the RPE. Currently, there is no practical formulation to overcome this issue. In this study, we demonstrated that simple mixing with adenosine tetraphosphate (ATP) has the potential to greatly enhance the transport and permeation of a polymeric nanocarrier across the retina via intravitreal administration. Chitosan-functionalized, pluronic-based nanocarrier (NC), which can deliver various biomolecules efficiently, was used as a polymeric nanocarrier. Mixing with ATP facilitated the diffusion of the nanocarrier in the vitreous humor by reducing the electrostatic interaction between NC and negatively charged glycosaminoglycans (GAGs) in the vitreous humor. Mixing with ATP also allowed the penetration of NC across the whole retina, and it resulted in a great increase (approximately nine times) in the transport of NC across the retina, as well as spreading it throughout the whole retina upon intravitreal administration in a mouse model. This enhanced permeation across the retina was specific to ATP but not to GTP, suggesting the possibility of P2Y receptor-mediated tight junction disruption by ATP.

## 1. Introduction

The human eye is a complicated part of our body. It consists of a two-piece unit, the anterior segment and the posterior segment. The anterior part of the eye is made up of the sclera, iris, conjunctiva, and cornea. The posterior part of the eye is made up of the vitreous humor, retina, choroid, and optic nerve. There are various barriers in each part of the eye to prevent reaching the therapeutic level of medicine in targeted tissues. In the anterior part of the eye, tear turnover, nasolacrimal drainage, and metabolism by esterase and protease are involved to reduce the concentration of administered drugs [1]. In the posterior eye segment, the retina layer acts as a major barrier against ophthalmic drug delivery. The retina is protected by the blood–retinal barrier (BRB), which maintains retinal homeostasis and shields the retina from the external stimulus [2,3]. The retina is the photosensitive component of the central nervous system [1], and the retina consumes a high level of oxygen while lining the innermost part. Therefore, the retina is susceptible to oxidative stress [3], which causes retinal diseases, including age-related macular degeneration (AMD), glaucoma, and diabetic retinopathy. Approaches for drug delivery to the posterior segment of the eye include systemic administration, modification of the barrier, and direct local injection (e.g., intravitreal injection). Topical and systemic administration is very inefficient for posterior ocular drug delivery, leading to almost no effect on the retina. Direct intravitreal injection is the most common and widely applicable route for posterior ocular drug delivery [4]. However, the injected medicine is subject to degradation by protease, and the viscous vitreous humor limits its diffusion to the retina. Furthermore, transport across the retina and retinal pigment epithelium (RPE) is very limited, especially for protein drugs (e.g., antibodies) [5]. These barriers require frequent administration by intravitreal injection and the frequent eye punctures during the intravitreal injection cause several side effects, including bleeding, retinal detachment, increased intraocular pressure (IOP) with damage to the optic nerve, and loss of vision [4]. Therefore, it is necessary to reduce the burden of intravitreal injection and improve the outcomes. In recent years, various strategies to deliver therapeutics into the posterior segment of the eye have been researched in the drug delivery area. One of them is the incorporation of therapeutic molecules into a hydrogel for targeted and sustained delivery with minimized toxicity [4,6]. For example, hydrogels that exhibit thermo-responsive behavior near body temperature such as poloxamer and poly(N-isopropyl acrylamide-co-acrylonitrile) (PNIPAAm) were studied for injection into the vitreous cavity. These polymers have a mucoadhesive property and optical clarity so that they could be successfully applied to the ocular drug delivery system [7]. However, no strategy to overcome the multiple barriers for targeted retina transport was provided. Various nanoparticles were studied to analyze the effect of the charge of the particle on the diffusion inside the vitreous body [8,9,10]. Anionic nanoparticles penetrated the vitreous barrier more efficiently than cationic nanoparticles because cationic nanocarriers could interact with the negatively charged vitreous glycosaminoglycan by electrostatic attraction. Furthermore, cationic nanoparticles did not penetrate the retinal structure. In contrast, anionic nanoparticles showed some penetrating ability across the retina to the RPE [11].

The RPE is a monolayer of pigmented cells, a part of the retina. The RPE constitutes the outer BRB with the tight junction [12]. The tight junction is a complicated structure that regulates cell polarity and paracellular diffusion of fluids and solutes [13], thus preventing the entrance of toxic molecules and plasma components into the retina. [14] On the RPE layer, there is a purinergic receptor [15]. The agonists against the purinergic receptor, especially ATP, have been reported to have a considerable impact on the RPE layer [14]. Because there is a P2Y receptor on the apical plasma membrane of the RPE, extracellular ATP can bind to the receptor. The P2Y receptor then reacts to the near actin cytoskeleton, filamin A, which induces the rearrangement of the RPE layer. [14] Through this effect, nucleotides were tried to induce the disassembly of the tight junction for increasing the transport across the RPE of therapeutics [16]. In particular, P1, P4-Di(adenosine-5′) tetraphosphate (AP_4_A) increased the corneal epithelial barrier’s permeability, and the P2Y receptor signaling pathway by its agonist was confirmed [17]. Furthermore, the P2Y receptor agonist was used to treat edematous retinal disorders as therapeutic and adjuvant agents [15]. In the clinic, the P2Y agonist nucleotide is used for retinal detachment [14].

**Figure 1:**
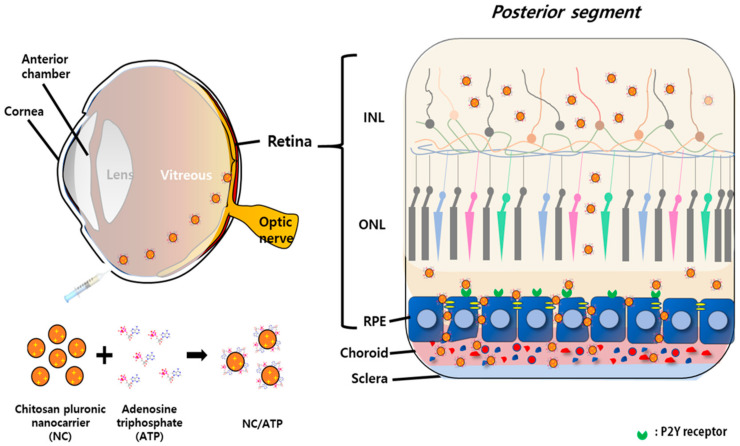
Strategy for transport through the retina with chitosan-functionalized pluronic-based nanocarrier (NC)–ATP. NC has a positive charge due to chitosan (marked as “+”) and ATP has a net negative charge. NC–ATP was simply prepared by mixing via electrostatic interaction. NC/ATP was intravitreally administered.

Chitosan-functionalized pluronic-based nanocarrier (NC) has been developed and applied for the delivery of proteins as an active state by us. Based on its thermosensitive, large-volume expansion property, a simple co-incubation with protein and temperature control resulted in a very high loading efficiency of various proteins [18]. In addition to its tumor-targeting and skin-penetration properties, additional functionalization with specific peptides could remarkably enhance the targeted delivery of loaded therapeutics. [11,19,20,21]. In this study, we applied this NC as a potential nanocarrier for therapeutic protein drugs toward the retina via intravitreal injection. As introduced in the preceding paragraph, positively charged nanosystems have difficulty in getting to the retina due to the interaction with hyaluronic acid in the vitreous humor. [11] Thus, to facilitate the transport through the vitreous humor by charge neutralization of a positively charged NC, and to enhance the transport of NC across the RPE by inducing the rearrangement of the tight junctions in the RPE layer, adenosine triphosphate (ATP), as a negatively charged nucleotide (NTP) and as an agonist against the P2Y receptor in the retina [12], was mixed with NC and the mixture was administered into the vitreous body of the eye (Figure 1). We hypothesized that the NC–ATP system could permeate through the retina layer by bypassing the tight junction in the RPE as well as the complicated vitreous environment upon intravitreal injection.

## 2. Materials and Methods

### 2.1. Materials

Pluronic F127 (PEOnPPOmPEOn, n = 100, m = 65) was a kind donation from BASF Corp. (Seoul, Korea). A cellulose ester dialysis bag (MWCO 300,000) was purchased from Spectrum (Houston, TX, USA). Water-soluble chitosan (Mw ~10 kDa; degree of deacetylation: 85%) was obtained from Amicogen (Seoul, Korea). Irgacure 2959 was purchased from Chiba Specialty Chemicals (Basel, Switzerland). Mono-reactive hydroxysuccinimide ester of Cy5.5 (Cy5.5-NHS) was obtained from GE Healthcare Biosciences (Pittsburgh, PA, USA). ZO-1 antibody, Alexa 633 secondary antibody, Alexa Fluor 594 phalloidin, and Dulbecco Modified Eagle Medium (DMEM, Gibco) were obtained from Thermo Fisher Scientific (Waltham, MA, USA). Nanosep centrifugal devices for spin-filtration (MWCO 300 kDa) were purchased from Pall Life Sciences (Ann Arbor, MI, USA). Adult retinal pigment epithelial cell line-19 (ARPE-19) cells were obtained from ATCC (Manassas, VA, USA). A Cell Counting Kit-8 (CCK assay kit) was obtained from Dojindo Molecular Technologies (Rockville, MD, USA). All other chemicals were purchased from Sigma-Aldrich (Saint Louis, MO, USA).

### 2.2. Preparation of Chitosan-Functionalized Pluronic-Based Nanocarrier

Chitosan-functionalized pluronic-based nanocarrier (NC) was synthesized by UV photo-crosslinking diluted diacrylated pluronic (DA-PF 127) and glycidyl methacrylated-chitosan (GMA-chitosan) with a photoinitiator (Irgacure 2959), as previously reported [18]. Briefly, diacrylated pluronic was prepared by reacting pluronic 127 (PF 127) with acryloyl chloride (the degree of substitution was 98% by NMR), then 0.77 wt% of DA-PF 127 and 0.14 wt% of GMA-chitosan were UV-irradiated for 15 min with Irgacure 2959 (0.05 wt%). The degree of substitution of GMA in GMA-chitosan was 15 wt%, which was measured by ^1^H-NMR. The size of NC at various temperatures was measured by dynamic light scattering (ELSZ, Otsuka Electronics, Osaka, Japan).

### 2.3. Cytotoxicity of NC

ARPE-19 (human retinal pigment epithelial cells) were seeded in a 96-well plate (5 × 10^3^ cells/well) and incubated overnight in a cell culture incubator at 37 °C. The culture medium (DMEM) from each well was then removed and replaced with fresh media containing various concentrations (0, 12.5, and 25 μg/mL) of NC. After 24 h, the culture supernatant was removed and a fresh medium containing 10% CCK assay reagent solution was added to each well. The plate was then incubated at 37 °C for 30 min, and the absorbance of the supernatant at 570 nm was measured using a microplate reader. All experiments were performed in triplicate. The percent viability was determined by the following equation.

(Abs_sample_ − Abs_control_)/(Abs_cell_ − Abs_control_) × 100 (%) (1)

### 2.4. Actin Rearrangement Effect of NC–NTP on the Retinal Cell Layer

ARPE-19 cells were seed in a 12-well plate (10 × 10^4^ cells/well) and cultured over 3 weeks to achieve a tightly bound monolayer mimicking the retinal pigment epithelium. First, the NC/ATP solution with 10 mg/mL of NC and 10 mM of ATP in De-ionized water (DIW) was prepared. Before treatment with NC or NC–NTP, the medium was removed, and the cell layer was washed with phosphate-buffered saline (PBS) buffer. Prepared NC or NC–NTP sample was incubated in 37 °C for 10 min; after that, 100 μL of the NC–ATP solution was treated into the well first and 1.9 mL of fresh DMEM was added into the sample-treated well. As control groups, NC alone or NC–GTP (as a non-agonist against the P2Y receptor) was also treated similarly. After 30 min of incubation at 37 °C, the supernatant medium was removed and washed with PBS. It was then stained using Alexa Fluor 594 phalloidin to visualize the actin filaments in the ARPE-19 cell. The result was obtained by confocal microscopy (Excitation: 594 nm, Emission: 570–573 nm).

### 2.5. Distribution of NC and NC–ATP in the Porcine Eye Ex Vivo

Fresh porcine eyes purchased from a local slaughterhouse were used for ex vivo experiments. Cy5.5, a fluorescent dye, was conjugated to NC (Cy5.5–NC) by reacting the amine groups of NC and Cy5.5-NHS, as previously reported [18] for optically monitoring NCs in the eye. The NC solution (100 μL) containing Cy5.5–NC (12 mg) or the NC–NTP solution (100 μL) containing Cy5.5–NC (12 mg) and 100 mM of ATP or GTP was injected into the vitreous humor of the porcine eye. After injection, the porcine eye samples in PBS were incubated for 24 h at 37 °C in a humidified cell culture incubator. They were then cut to monitor the middle part of the eye sphere. The distribution of Cy5.5–NC in the eye was observed by fluorescence using a fluorescence imaging system (FOBI, NeoScience, Seoul, Korea).

### 2.6. Retention of NC in the Eye and Permeation through the RPE to the Blood Vessels in Mice

Eight-week-old BALB/C male mice from Orient Bio Inc. (Seongnam, Korea) were used for in vivo experiments. All experiments were carried out according to the guidelines of the animal care and use committee of the Gwangju Institute of Science and Technology (GIST) (GIST-2018-068). Mice were randomly divided into 6 groups with 3 mice per group (*n* = 3). The mice were fully anesthetized by isoflurane and the corneas were dilated using phenylephrine and atropine drops. Approximately 6 μL of the solution containing 10 mg/mL Cy5.5–NC or Cy5.5–NC–NTP (100 mM) was injected into the vitreous humor of the eye. After 6 h of intravitreal injection, fluorescence imaging of the injected eye was obtained by using a fluorescence imaging system (FOBI). At 6 h after injection of the sample solution, the blood was collected from the mouse eye (retro-orbital plexus). The collected blood was centrifuged at 4 °C and 3000 rpm for 5 min to obtain the serum. The fluorescence from the serum was measured by a multiwall microplate reader (Thermo Fisher Scientific, Waltham, MA, USA).

### 2.7. Histogolical Analysis of NC–NTP in the Retina

At 6 h after injection of Cy5.5–NC or Cy5.5–NC–NTP, the eyes were collected after sacrificing the mouse and fixed in 4% formalin solution for 1 h. Each eye was then embedded in the optimal cutting temperature (OCT) compound, frozen at −20 to −30 °C and cryo-sectioned to 5 μm thickness to monitor the distribution of Cy5.5-NC in the retina by confocal microscopy. The sectioned eye samples were stained by 4′,6-diamidino-2-phenylindole (DAPI) to visualize the retina structure.

Immunofluorescence staining of the tight junction was performed in the retinal pigment epithelium (RPE) layer. After 24 h of intravitreal injection, the sample-injected eyes were opened by circumferential incision at the surgical limbus, and the anterior segment and lens were removed. The posterior segment of the eye was put on filter paper and fixed in a 4% formalin solution for 2 h. After fixation, it was possible to separate the posterior segment of the eye from the filter paper while retaining a flat structure. This flat structure was stained with ZO-1 primary antibody diluted with 1% bovine serum albumin (BSA) blocking solution. (1:100). The stained section was visualized with Alexa 633 secondary antibody and observed under fluorescence microscopy.

### 2.8. Statistical Analysis

All values are expressed as means ± standard deviation. Differences between groups were examined for statistical significance by the two-tailed student *t*-test using Microsoft Excel. A *p*-value of ≤0.05 was considered to be significant, with the symbols **: *p* < 0.01, *: *p* < 0.05, and #: *p* > 0.05.

## 3. Results and Discussion

### 3.1. Biocompatibility of NC and Bioactivity of ATP-Mixed Chitosan-Functionalized Pluronic-Based Nanocarrieron the Retinal Pigmented Epithelium

**Figure 2:**
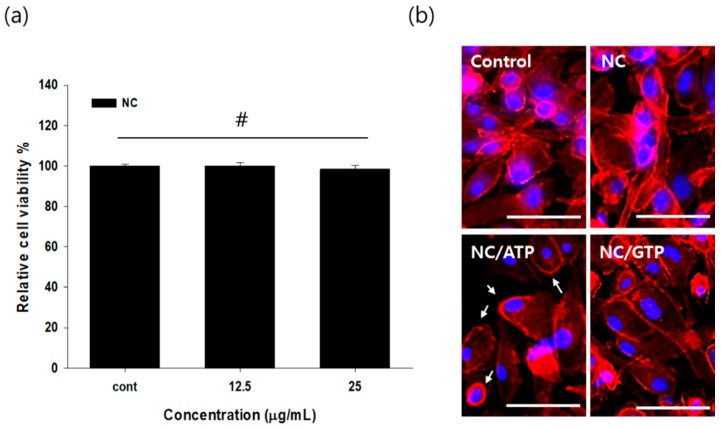
Biocompatibility of NC and rearrangement of the cytoskeleton of ARPE-19 after treatment with NC–ATP. (**a**) Metabolic activity of ARPE-19 measured by a Cell Counting Kit-8 (CCK) assay after 24 h of treatment with NC. #: No significant difference by *t*-test with *p* > 0.05. (**b**) Representative images of F-actin staining of ARPE-19 with phalloidin Alexa-594 (red). Blue: Nuclear staining with DAPI. The observed round cell morphology is indicated by arrows (scale bar: 100 μm).

The biocompatibility of chitosan-functionalized pluronic-based nanogel (NC) on fibroblast cells and colon epithelial cells was previously verified by us [20,21]. In this study, we further evaluated the cytotoxicity of NC at various concentrations on ARPE-19, human retinal pigment epithelium cells. As expected, NC did not show any cytotoxic effect on ARPE-19 up to 25 μg/mL, showing a similar metabolic rate of the cells compared with the control cell-only group by the CCK assay kit (Figure 2a).

After mixing ATP and NC to form NC–ATP, NC–ATP was administered to the ARPE-19 cell layer for 30 min. The ARPE-19 cell layer was then stained with phalloidin dye to see the cells’ morphology. Because the P2Y receptor is linked to the actin cytoskeleton, the P2Y receptor agonist is known to initiate cytoskeletal rearrangements [14,22]. As shown in Figure 2b, some reduction in actin filament stretching, leading to the round morphology of cells, was observed in the NC–ATP compared with the control, the NC group, or even the NC–GTP group. It was reported that ATP can induce cell rearrangement associated with the P2Y receptor [23,24,25]. Thus, this result suggested the activity of ATP in NC–ATP in the rearrangement of ARPE-19, possibly mediated by the P2Y receptor.

### 3.2. Distribution of NC and NC–NTP in the Porcine Eye Ex Vivo

**Figure 3:**
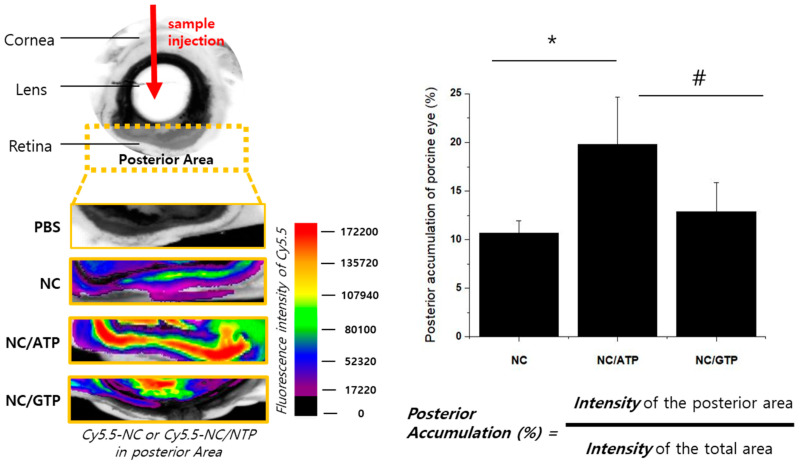
Ex vivo Cy5.5–NC or Cy5.5–NC–negatively charged ATP (NTP) distribution in porcine eyes and accumulated amount on the posterior area (*n* = 3). The fluorescence of Cy5.5 was measured by a fluorescence imaging system. Statistical analysis by *t*-test: *: *p* ≤ 0.05; #: *p* > 0.05.

The pig eye is often used as an ex vivo model in eye-related research because of its similarity to the human eye [4]. The distribution of NC or NC–NTP was analyzed upon intravitreal injection (Figure 3). After 24 h of incubation at 37 °C, the eye was dissected across the whole eye including the cornea, vitreous body, retina, and choroid/sclera. The fluorescence signal of Cy5.5–NC showed a significantly larger accumulation of NC in the posterior part for NC–ATP than NC alone. NC–ATP also showed a larger accumulation in the posterior part even compared with NC–GTP, although the difference was not statistically significant due to the large variations among samples. The larger accumulation of NC in the posterior part by the addition of ATP supported the efficient and positive role of ATP for diffusion throughout the vitreous body by reducing electrostatic interaction of positively charged NC with the negatively charged vitreous glycosaminoglycans (GAGs) (mainly hyaluronic acid). On the other hand, the tendency to show better accumulation of NC–ATP in the posterior part than NC–GTP also suggested the potential role of the P2Y receptor in the retina for targeted delivery to the retina by the interaction with the P2Y receptor, considering that both GTP and ATP have the same charge state and a similar structure (pyrimidine base), whereas ATP is an agonist against the P2Y receptor but GTP is not. However, the limitation of this ex vivo result should be noted: since no physiological efflux system was available in the eye, the result does not imply that more accumulation in the retina would occur in vivo by the addition of ATP, but the transport and penetration of NC to the retina might be enhanced by ATP.

### 3.3. Retention of NC–NTP in the Eye and Permeation of NC–NTP across the Retina In Vivo

**Figure 4:**
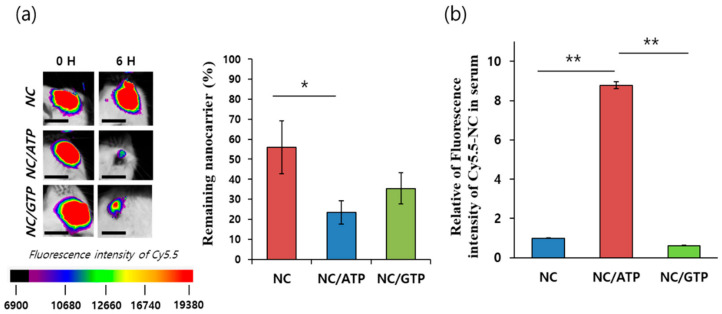
In vivo (**a**) retention of Cy5.5–NC or Cy5.5–NC–ATP in the eyes by fluorescence intensity at 6 h compared with the initial values (scale bar: 3 μm), and (**b**) permeated Cy5.5–NC or Cy5.5–NC–ATP in the serum 6 h after intraocular administration (*n* = 3). Statistical analysis by *t*-test. *: *p* ≤ 0.05; **: *p* ≤ 0.01.

After intravitreal administration of NC–NTP, retention of Cy5.5–NC in the eye was monitored by a fluorescence imaging system. At 6 h after injection, still more than half (56%) of the initially injected NC remained in the eye. In contrast, NC–ATP showed a substantially lower (23%) amount remaining in the eye. NC–GTP also showed a similar tendency of a reduced amount (35%). The remaining amount of NC/GTP was a little higher than that of NC/ATP, but the difference was not statistically significant (Figure 4a). This result seemed to be the opposite of the ex vivo results in Figure 3. However, in the ex vivo experiment, we analyzed the remaining sample in the posterior part of the whole eye during culture. Thus, the in vivo result obtained here showed more efficient efflux or clearance of the injected NC from the vitreous body to the outside of the eye by ATP or GTP, supporting the facilitated diffusion of NC inside the vitreous body. Various clearance routes for injected NC are available, so to analyze the penetration of NC across the retina, the fluorescence intensity of Cy5.5–NC in the serum was analyzed. As shown in Figure 4b, approximately nine times higher intensity of Cy5.5–NC was observed from NC–ATP compared with NC alone. This result strongly supported the efficient and positive role of ATP on the enhanced permeation of NC across the retina upon intravitreal injection. Interestingly, NC–GTP did not show any improvement compared with NC alone. Thus, although GTP is considered to be effective in facilitated diffusion inside the vitreous body, similar to ATP, it was not effective at all to promote the permeation of NC across the retina. The interaction between the P2Y receptor and ATP but not GTP has been reported [26,27,28]. This result suggests the potential role of the P2Y receptor in enhancing the permeation of NC across the retina. The nanocarrier could infiltrate into the choroid/sclera by the rearrangement of the retinal pigment epithelial cell layer with the P2Y receptors, leading to more efficient flow out to the blood in the choroid. [6] Based on the assumption of ~1 mL of total circulating blood volume in a mouse, the amount of NC in the blood was estimated to be significant, ~6.7% of the initially injected amount for NC–ATP.

### 3.4. Histological Analysis of NC–NTP in the Retina

**Figure 5:**
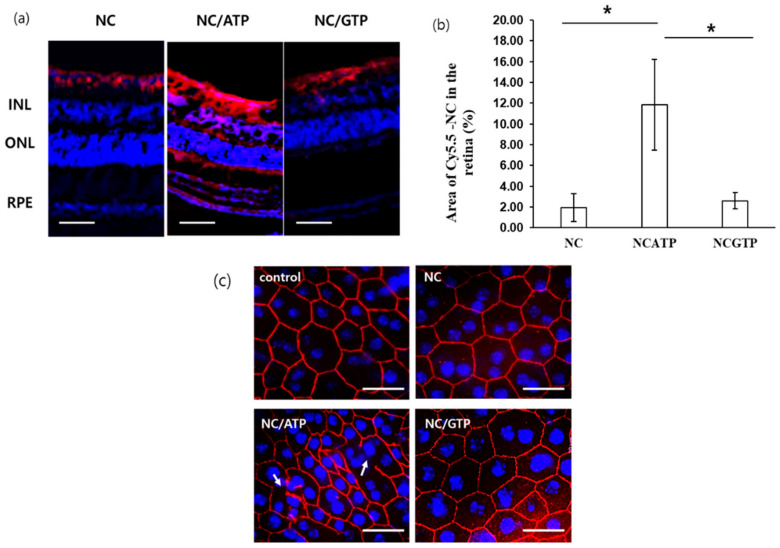
(**a**) In vivo penetration of Cy5.5–NC (red) or Cy5.5–NC–NTP across the retina 6 h after intravitreal injection (scale bar: 50 mm). INL: inner nuclear layer; ONL: outer nuclear layer; RPE: retinal pigment epithelium. Nuclear staining by DAPI (blue). (**b**) Semi-quantitative analysis of Figure 5a showing the penetrated nanocarriers in the retina. Statistical analysis by *t*-test. *: *p* ≤ 0.05 (**c**) ZO-1 staining (red) of the retina 6 h after intravitreal injection of NC or NC–NTP (scale bar: 20 mm). Nuclear staining by DAPI (blue).

The retina at 6 h after injection was sectioned and analyzed by fluorescence microscopy (Figure 5a). Red fluorescence from Cy5.5–NC and blue fluorescence from retinal cell nuclei stained with DAPI were visualized. In the case of NC alone, some NC was observed above the inner nuclear layer (INL), showing that some of NC could reach the surface of the retina, but almost no NC was observed throughout the retina, implying no evidence of permeation across the retina. In contrast, a much stronger red signal of Cy5.5–NC–ATP was observed throughout the whole retina section. First, the area of the red signal on the surface of the retina was much stronger and larger than that of NC alone, implying more efficient diffusion throughout the vitreous body by ATP addition, consistent with other data. Red fluorescence was also observed in the whole retina including the INL, outer nuclear layer (ONL), and RPE, where the P2Y receptors are located [29]. This result showed direct evidence of the permeation of NC across the retina. In the case of NC–GTP, red fluorescence was also observed on the surface of the retina, supporting the positive role of GTP for diffusion throughout the vitreous body by reducing electrostatic interaction between positively charged NC and the vitreous body containing anionic GAGs. However, a much lower amount of red fluorescence was observed inside the retina. A semi-quantitative analysis of Cy5.5–NC signals using sectioned images showed an approximately six times higher density of Cy5.5–NC in the NC–ATP group than in the NC group or NC–GTP group in the retina (Figure 5b), coinciding well with the results of the serum concentration of NC in Figure 4b. The clear difference between NC–ATP and NC–GTP suggested the involvement of the P2Y receptor in the permeation of NC across the retina.

Immunocytochemical analysis was performed on the mouse retina to detect the tight junctions in the RPE with ZO-1. As shown in Figure 5c, the control, NC alone, and NC–GTP groups showed well-developed tight junction distribution. This result implies that the intravitreal sample injection in this experiment did not severely damage the retina to disrupt the RPE layer. In contrast, some disruption in the tight junction was observed only in the NC–ATP group, supporting the P2Y receptor-mediated rearrangement of the RPE cells, allowing the permeation of NC across the RPE.

In the route of intravitreal injection, there are several anatomical and physiological barriers such as the vitreous, inner limiting membrane (ILM), cellular binding, extracellular matrix, and efflux transporters [16,24,25,26]. Even though the development of nanosystems for ocular drug delivery has not been practically applied yet, several nanoparticle systems have shown promising results in terms of biocompatibility, biodegradability, non-immunogenicity, extended half-life of drugs, easy engineering, and enhanced tissue penetration [11,26,27]. Most of these studies using nanosystems for drug delivery to the retina are intended to diffuse well throughout the vitreous body and permeate across the retina to reach the RPE. Hyaluronic acid (HA), the major constituent of the vitreous body, has a negative charge and binds to the cell surface receptor, including CD44 in the retina. Thus, the HA-modified nanosystem showed significantly rapid diffusion throughout the vitreous body and reached the retina specifically, not other tissues [9]. A PEG-coated nanoparticle system with no immune response reached the retina to induce better treatment than the free drug [27]. A PLGA-based nanosystem improved the bioavailability and decreased the side effects of a free drug, resulting in over two times the anti-angiogenic effect on the retina compared with the free drug [28]. However, all of these studies did not show quantitative comparisons with the control groups in terms of penetration of the carriers to prove their strategy. On the contrary, we showed remarkably enhanced transport and permeation of a polymeric nanocarrier with quantitative analyses by premixing it with ATP in this study, and the estimated amount of permeated nanocarrier in the serum was significant. However, we did not load any drug into the nanocarrier or characterize the treatment effect in this study. Therefore, the evaluation of drug-loaded nanocarriers using a retina-disease animal model is necessary to prove the effectiveness of the present approach.

## 4. Conclusions

Mixing ATP with NC, a polymeric nanocarrier, resulted in a great increase (approximately nine times) in the transport of NC across the retina as well as spreading it throughout the whole retina upon intravitreal administration. ATP might have contributed to this enhancement by two factors. First, negatively charged ATP facilitated the diffusion of NC throughout the vitreal humor by reducing the electrostatic interaction between positively charged NC and negatively charged GAGs (mostly hyaluronic acid) in the vitreous humor. Second, ATP enhanced the penetration of NC into the retina, potentially mediated by the interaction with the P2Y receptors. The involvement of the P2Y receptor in the enhanced penetration of NC was supported by the specificity of ATP in contrast to GTP and tight junction disruption. If we consider that a significant amount of the injected NC could permeate through the RPE, the strategy used in this study has high potential applicability for important retina-associated diseases such as diabetic retinopathy (DR) or proliferative age-related macular degeneration (AMD) that require the targeted delivery of therapeutic drugs to the retina for treatment or imaging agents for monitoring the progress of the diseases.

## Data Availability

The data presented in this study are available in the research article.
